# An Unusual Presentation of Morphea: A Case Report

**DOI:** 10.7759/cureus.73614

**Published:** 2024-11-13

**Authors:** Asiya Al Badi, Maryam Al-Khamisani

**Affiliations:** 1 General Practice, Directorate of Primary Health Care, Al Buraimi, OMN; 2 Dermatology, Al Buraimi Hospital, Al Buraimi, OMN

**Keywords:** corrugated cords, female, linear papules, morphea, neck

## Abstract

Morphea is a chronic inflammatory skin disease characterized by skin fibrosis with variable clinical presentation. We report a case of a young woman who presented with asymptomatic progressive indurated short cords on the neck. A diagnosis of morphea was made based on clinical and histopathological findings. She had clinical improvement after starting topical corticosteroids alternating with topical tacrolimus.

## Introduction

Morphea is a chronic inflammatory connective tissue disorder occurring in adults and children, with an incidence of 4-27 cases per million people per year worldwide [1]. The pathogenesis of morphea is influenced by genetic factors, environmental elements (such as infections and trauma), and immune system imbalances [1]. It progresses through three stages: 1) initial inflammation, characterized by the infiltration of immune cells and the release of cytokines; 2) fibrosis, where stimulated fibroblasts generate excessive collagen; and 3) atrophy, which is characterized by thinning of the tissue and a decrease in sclerosis [1]. It is characterized by fibrosis with variable clinical presentations and is divided into five main types: limited, generalized, linear, deep, and mixed [1]. Morphea clinically presenting as indurated short cords is rarely reported in the literature [2]. Systemic sclerosis (SSc) is an autoimmune disease characterized by skin fibrosis and involvement of internal organs. Systemic sclerosis can be distinguished from morphea by facial features like telangiectasia and a beak-shaped nose, as well as vascular symptoms such as Raynaud's phenomenon and digital ulcers [3]. Additionally, specific serum antibodies like anti-centromere and anti-Scl-70 antibodies are present in SSc but absent in morphea [3]. 

We report a case of an unusual presentation of morphea and review the clinical, histopathological presentation and treatment of this case.

This article was presented as an e-poster at the 19th Symposium of the European Academy of Dermatology and Venereology in Malta on 16-18 May 2024.

## Case presentation

A 24-year-old woman presented to the dermatology clinic with an insidious onset of multiple asymptomatic skin-colored papules on the anterior neck of many years' duration. She had no history of joint pain, oral ulcers, or photosensitivity. There was no significant past medical, family history, allergy, or drug history. No personal or family history of autoimmune diseases and no history of trauma or insect bite were present.

**Figure 1:**
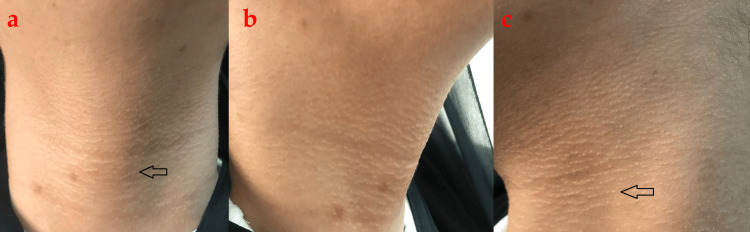
On presentation, papules forming short linear cords on the anterior neck (a; black arrow), extending laterally on the right side (b) and the left side (c; black arrow)

On examination, there were multiple skin-colored tiny linear papules forming short linear cords on the anterior neck, extending laterally on both sides. The lesions were arranged horizontally and have variable lengths (Figure 1). No similar lesions were found elsewhere on the body or flexural areas. There were no signs suggesting systemic sclerosis, such as abnormal nail fold capillaries, no sclerodactyly, distal digital pitting/ulcers, or the Raynaud phenomenon. 

Basic laboratory tests were within normal limits, which included routine hematological tests, kidney function tests, and liver function tests. Further laboratory tests were not done as the patient had no other associated symptoms or systemic manifestations.

Before the skin biopsy, we considered various differential diagnoses such as systemic sclerosis, pseudoxanthoma elasticum, and mucin deposition diseases.

**Figure 2:**
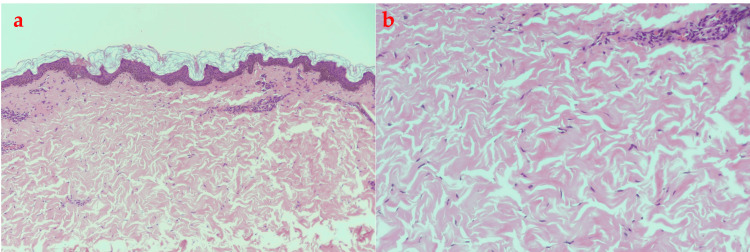
(a) Hematoxylin and eosin-stained skin biopsy (20x magnification) showing thinned out epidermis, the dermis showed thickened eosinophilic collagen bundles in the papillary and reticular dermis along with perivascular lymphocytic inflammation; (b) A higher magnification showing collagen bundles (40x magnification)

The biopsy showed thinned-out epidermis, and the dermis showed thickened eosinophilic collagen bundles in the papillary and reticular dermis along with perivascular lymphocytic inflammation (Figure 2). Alcian blue stain showed mild mucin deposition between the collagen bundles. 

**Figure 3:**
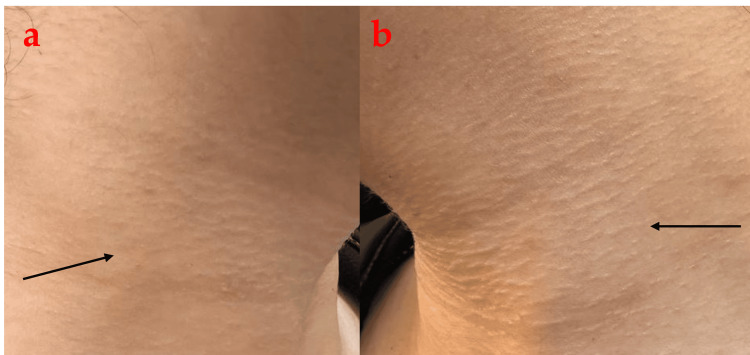
After two months of treatment, the right side of the neck (a) showing noticeable flattening of the lesions as shown by the black arrow. The left side of the neck (b) showed mild flattening of the lesions (black arrow)

Based on these clinical and pathological findings, the diagnosis of morphea was made. The patient was started on mometasone furoate cream 0.1 % once daily and tacrolimus ointment 0.1% at bedtime. After two months of treatment, the patient showed noticeable improvement (Figure 3), and no new lesions developed. Examination of the neck revealed flatter lesions on both sides as compared to the midline. She was advised to continue the same treatment but to reduce topical steroids to twice weekly and continue topical tacrolimus on other days. After a three-month follow-up appointment, the patient had no further progression and was advised to continue the same treatment. 

## Discussion

This report describes a young female who presented with multiple papules forming short linear cords on the neck. The clinical distribution of these lesions was unusual. The biopsy results confirmed the presence of histopathological features consistent with localized scleroderma.

A literature review was conducted in PubMed and Google Scholar. We used the terms 'morphea', 'elevated cords', 'indurated cords', 'anterior neck', and 'lateral neck'. To the best of our knowledge, this is only the second case reported with these features [2].

The presentation of raised lesions in morphea is rare. Nodular morphea [4] and cord-like [5] variants are rare variants of morphea that present as elevated lesions. Ohata et al. reviewed nineteen nodular morphea cases, in which most cases presented with 0.5 to 3 cm nodules commonly seen on the chest and back and less commonly on the upper extremities [4]. Two cases reported by Tomasini [5] showed elevated cord-like lesions on the sides of the chest. An-Kang Gu et al. [2] also reported a unique manifestation of morphea presented as corrugated indurated short cords on the neck and upper chest, which is similar to our patient's presentation but to a lesser extent.

These indurated linear cords resemble pseudoxanthoma elasticum, which typically presents as small, asymptomatic, yellowish, or skin-colored papules that coalesce into plaques on the neck and the flexural areas [6]. The histological examination of pseudoxanthoma elasticum usually shows fragmented elastic fibers and mid-dermal calcification, which can be identified using Verhoeff-Van Gieson stain for fragmentation and von Kossa or Alizarin Red stains for calcification [6], which is not fitting with the histopathological findings of our patient's biopsy. Moreover, she lacked ocular and cardiovascular manifestations. These lesions might also resemble mucin deposition disorders like lichen myxedematosus, which clinically presents as waxy, firm papules and nodules arranged in a linear array and histologically as mucin deposition, proliferation of fibroblasts, and increased collagen deposition, which are not fitting with the histological findings of our case [7]. Moreover, elevated cords may resemble scleromyxedema and hypertrophic scar tissue. Differentiation of these conditions can be achieved by histopathological examination, which reveals the distinct characteristics of each. Scleromyxedema manifests as a triad of mucin, fibroblast proliferation and fibrosis, and an interstitial granuloma annulare-like pattern [8]. Hypertrophic scar tissue histopathological findings are collagen bundles are flatter and arranged in a wavy pattern but predominantly oriented parallel to the epithelial surface [9]. The above histopathological findings of the differential diagnoses were different that the patient's histopathological findings.

In this case, the disease was limited, with no signs of systemic involvement. Hence, we decided to initiate topical treatment. The patient showed a clinical response within the first two months of use without further disease progression. She continued to improve in the following follow-up appointment and was monitored for any potential disfigurement or systemic involvement.

## Conclusions

In conclusion, indurated corrugated short cords of anterolateral neck are an unusual presentation of morphea. An accurate diagnosis is essential for appropriate treatment. The limited disease responds well to topical steroids alternating with topical tacrolimus. Further research is needed to understand this uncommon presentation of morphea and its underlying mechanism.

## References

[1] Papara C, De Luca DA, Bieber K, Vorobyev A, Ludwig RJ (2023). Morphea: the 2023 update. Front Med (Lausanne).

[2] Gu AK, Li J, Zhang LT (2021). Corrugated indurated short cords on the neck of a teenage girl. JAMA Dermatol.

[3] Knobler R, Moinzadeh P, Hunzelmann N (2017). European Dermatology Forum S1-guideline on the diagnosis and treatment of sclerosing diseases of the skin, Part 1: localized scleroderma, systemic sclerosis and overlap syndromes. J Eur Acad Dermatol Venereol.

[4] Ohata C, Yasunaga M, Tsuruta D (2013). Nodular morphea (NM): report of a case of concurrent NM and morphea profunda associated with limited type systemic sclerosis, and overview and definition for NM. Eur J Dermatol.

[5] Tomasini C (2016). Cordoniform morphea: a clinicopathologic study of two cases presenting with the rope sign. J Cutan Pathol.

[6] Marconi B, Bobyr I, Campanati A (2015). Pseudoxanthoma elasticum and skin: clinical manifestations, histopathology, pathomechanism, perspectives of treatment. Intractable Rare Dis Res.

[7] Cárdenas-Gonzalez RE, Ruelas ME, Candiani JO (2019). Lichen myxedematosus: a rare group of cutaneous mucinosis. An Bras Dermatol.

[8] Rongioletti F, Merlo G, Carli C (2016). Histopathologic characteristics of scleromyxedema: a study of a series of 34 cases. J Am Acad Dermatol.

[9] Wolfram D, Tzankov A, Pülzl P, Piza-Katzer H (2009). Hypertrophic scars and keloids - a review of their pathophysiology, risk factors, and therapeutic management. Dermatol Surg.

